# Bone marrow mesenchymal stem cell-derived exosomal miR-30e-5p ameliorates high-glucose induced renal proximal tubular cell pyroptosis by inhibiting ELAVL1

**DOI:** 10.1080/0886022X.2023.2177082

**Published:** 2023-02-16

**Authors:** Jia Lv, Ya-Ning Hao, Xiao-Pei Wang, Wan-Hong Lu, Li-Yi Xie, Dan Niu

**Affiliations:** Department of Nephrology, College of Medicine, Nephrotic Hospital, First Affiliated Hospital, Xi’an Jiaotong University, Xi’an, PR China

**Keywords:** Pyroptosis, bone marrow mesenchymal stem cells, exosome, miR-30e-5p, diabetic kidney disease

## Abstract

**Background:**

The rapid increase in the prevalence of diabetes has resulted in more cases of diabetic kidney disease (DKD). Treatment with bone marrow mesenchymal stem cells (BMSCs) may represent an alternative strategy to manage DKD.

**Methods:**

HK-2 cells were treated with 30 mM high glucose (HG). Bone marrow MSC-derived exosomes (BMSC-exos) were isolated and internalized into HK-2 cells. 3-(4,5-Dimethylthiazol-2-yl)-2,5-diphenyltetrazoliumbromide (MTT) and lactate dehydrogenase (LDH) assays were used to measure viability and cytotoxicity. The secretion of IL-1β and IL-18 was measured by ELISA. Pyroptosis was assessed by flow cytometry. Quantitative RT-PCR was used to measure the levels of miR-30e-5p, ELAV like RNA binding protein 1 (ELAVL1), IL-1β, and IL-18. The expression of ELAVL1 and pyroptosis-associated cytokine proteins was determined by western blot analysis. A dual-luciferase reporter gene assay was conducted to confirm the relationship between miR-30e-5p and ELAVL1.

**Results:**

BMSC-exos decreased LDH, IL-1β, and IL-18 secretion and inhibited the expression of the pyroptosis-related factors (IL-1β, caspase-1, GSDMD-N, and NLRP3) in HG-induced HK-2 cells. Moreover, miR-30e-5p depletion derived from BMSC-exos promoted HK-2 cell pyroptosis. Besides, miR-30e-5p over-expression or ELVAL1 knockdown could directly inhibit pyroptosis. ELAVL1 was a target of miR-30e-5p and knocking down ELAVL1 reversed the effect of miR-30e-5p inhibition in BMSC-exos-treated HK-2 cells.

**Conclusions:**

BMSC-derived exosomal miR-30e-5p inhibits caspase-1-mediated pyroptosis by targeting ELAVL1 in HG-induced HK-2 cells, which might provide a new strategy for treating DKD.

## 1. Introduction

Diabetic kidney disease (DKD) can cause chronic kidney disease. DKD is associated with albuminuria, progressive loss of kidney function, glomerular hypertrophy, and renal fibrosis [1,2]. The conventional treatment for DKD is effective in the early stages of renal damage, but not at an advanced stage, in which hyperglycemia and hypertension occur [3]. Dialysis and kidney transplantation are also helpful in treating kidney failure, but immune system rejection and a lack of donors are the primary limitation for their overall success [4]. Therefore, it is necessary to identify the mechanism of DKD in detail to develop new treatment strategies.

With the development of stem cell therapy, mesenchymal stem cells (MSCs) have gained interest as a potential therapy for renal damage [5]. MSCs exhibit a self-renewal capacity and multilineage differentiation potential [3]. Bone marrow, adipose tissue, and umbilical cord are the primary sources of MSCs. MSCs have protective effects against diabetic retinopathy, myocardial infarction, diabetes, and DKD [4,6–8]. In addition, MSCs can secrete cytokines, chemokines, growth factors, and extracellular vesicles [9]. Exosomes, which are a type of extracellular vesicle, range in size from 30 to 150 nm and are ubiquitous in body fluid. The underlying mechanism of bone marrow MSC-derived exosomes (BMSC-exos) may involve their function as mediators of intercellular communication and immune regulation [10,11].

Exosomes contain mRNAs, microRNAs (miRNA), and proteins. miRNAs are small noncoding transcripts that can regulate cell proliferation, apoptosis, oxidative stress, inflammatory factors, as well as other functions and participate in the occurrence and development of tumors, diabetes, Parkinson’s disease, and other diseases [12]. Recently, the effects of miRNAs on kidney disease have attracted attention. Thus far, miRNA dysregulation was shown to cause DKD, indicating that miRNAs may be useful diagnostic biomarkers for DKD [13]. miR-30e-5p expression was dysregulated in liver carcinoma, chronic myeloid leukemia, lung adenocarcinoma, colorectal cancer, and type 1 diabetes [14–17]. In addition, miR-30e-5p was predicted to be highly expressed in BMSC-exos; however, it was down-regulated in DKD [18]. Thus, we hypothesized that miR-30e-5p may be effective in regulating HG induced renal tubular epithelial cell injury.

Pyroptosis refers to the process of pro-inflammatory programmed cell death [19]. The NOD-like receptor pyrin containing receptor 3 (NLRP3) inflammasome, ELAV-like RNA binding protein 1 (ELAVL1), and caspase-1 can promote the secretion of inflammatory cytokines and cause plasma membrane rupture, which are considered hallmark features of pyroptosis [20,21]. Moreover, ELAVL1 could be targeted by miR-23c to regulate HG induced renal tubular epithelial pyroptosis [22]. HuR, a member of the ELAV protein family, contributes to ischemia, renal fibrosis, matrix protein accumulation, and angiogenesis. Furthermore, HuR was found to be up-regulated in DKD patients; thus, HuR may play an important role in the pathogenesis of DKD [23]. It was proved that pyroptosis participated in the process of DKD and NLRP3 inflammasomes promoted DKD progression [24,25]. The starBase database (http://starbase.sysu.edu.cn) predicted that ELAVL1 may be a downstream target gene of miR-30e-5p, but such a relationship has not yet been reported.

In the present study, we hypothesized that bone marrow mesenchymal stem cell (BMSC)-derived exosomal miR-30e-5p ameliorates HG-induced HK-2 cell pyroptosis by targeting ELAVL1. Our findings may provide a new strategy for the treatment of DKD.

## 2. Materials and methods

### 2.1. Cell culture and drug treatment

HK-2 (human renal proximal tubular cells) cells were obtained from the American Type Culture Collection (Manassas, VA). The cells were cultured in RPMI 1640 media containing 10% FBS, hydrocortisone (5 ng/ml), glutamine (2 mM), sodium selenite (5 ng/ml), insulin (5 μg/ml), transferrin (5 μg/ml), penicillin (100 U/ml), and streptomycin (100 μg/ml) in 5% CO_2_/95% humidified air at 37 °C. To simulate a DKD model *in vitro*, 30 mM glucose was added for 72 h to establish the HG model, whereas 5.5 mM glucose was added as a normal (NG) control and an additional 24.5 mM mannitol was added as an isotonic control [17].

### 2.2. Preparation and characterization of BMSC-exos

BMSC-exos were obtained from the supernatant of human-BMSCs and collected at a density of 1.0 × 10^5^ cells/well in a six-well plate. They were centrifuged at 2000×*g* for 20 min. The conditioned medium was ultracentrifuged at 100,000×*g*. Exosomes were washed once with 4-(2-hydroxyethyl)-1-piperazineethanesulfonic acid (HEPES) (Sigma-Aldrich, St. Louis, MO) and the above procedure was repeated. The exosomes were resuspended in PBS, filtered, and stored at −80 °C. Transmission electron microscopy was used to detect the presence of BMSC-exos and the particle sizes were assessed by nano-particle tracking analysis (NTA). The surface markers CD73, CD90, CD105, CD34, CD14, and CD45 were detected by Fluorescence-activated Cell Sorting (FACS) analysis. The surface markers CD63 and TSG101 were also analyzed by western blot analysis. Exosomes (100 μg/ml) were tagged with PKH67, a fluorescent linker dye (Sigma-Aldrich, St. Louis, MO), to trace them within cells. Finally, 30 μg BMSC-exos were added to HK-2 cells to determine their effect [26].

### 2.3. Internalization of BMSC-exos in HK-2 cells

BMSC-exos were labeled with the PHK67 membrane dye and 100 μg/ml were added to 3 × 10^4^ HK-2. The cells were then washed with PBS and fixed with paraformaldehyde. The nucleus was counterstained with DAPI (1:200) and observed by fluorescence microscopy (Nikon, Tokyo, Japan) [27].

### 2.4. 3-(4,5-Dimethylthiazol-2-yl)-2,5-diphenyltetrazoliumbromide (MTT) assay

Cells were seeded into 96-well plates at a density of 2000 cells per well. After a 24-h incubation, the medium was removed and MTT (5 mg/ml, M2003, Sigma-Aldrich, St. Louis, MO) was added to each well. After 3 h, the MTT was removed and 200 μl of DMSO were added to each well. The absorbance was measured at 490 nm using a microplate reader (Tecan, Mannedorf, Switzerland).

### 2.5. Flow cytometry

HK-2 cells were plated in a six-well plate at a density of 2 × 10^5^ cells per well. After 48 h, the cells were harvested, washed with PBS, and centrifuged at 1500 rpm for 5 min. FAM-YVAD-FMK (FAM600-1, Cell Technology, Hayward, CA), caspase-1 antibody, and propidium iodide were added to the resuspended cells and mixed. After incubating at room temperature in the dark for 1 h, the cells were analyzed using a BD Biosciences FACS Calibur system (San Jose, CA).

### 2.6. Cell transfection

The miR-30e-5p inhibitor (5′-CUUCCAGUCAAGGAUGUUUACA-3′) and negative control (inhibitor NC, 5′-CAGUACUUUUGUGUAGUACAA-3′), miR-30e-5p mimics (sense: 5′-UGUAAACAUCCUUGACUGGAAG-3′, anti-sense: 5′-CUUCCAGUCAAGGAUGUUUACA-3′) and negative control (mimics NC, sense: 5′-UUCUCCGAACGUGUCACGUTT-3′, anti-sense: 5′-ACGUGACACGUUCGGAGAATT-3′) and shRNA sequence targeting ELVAL1 (shELVAL1, 5′CGTGGATCAGACTACAGGTTT-3′) and negative control (shNC, 5′-GTTCTCCGAACGTGTCACGT-3′) were synthesized by GenePharma (Shanghai, China). Lipofectamine 2000 (Thermo Fisher, Waltham, MA) was used for plasmid and siRNAs transfection. After 48–72 h transfection, the cells were harvested for additional assays.

### 2.7. Dual-luciferase reporter gene assay

Cell transfection was done as described above. Briefly, ELAVL1 3′-UTRs containing miR-30e-5p binding sites (ELAVL1-WT: 5′-GUUUAC-3′) and its mutant (ELAVL1-MUT: 5′-CAAAUG-3′) were constructed and cloned into the pGL3 vector. The recombinant luciferase vector and mimics-miR-30e-5p/mimics NC were co-transfected into HK-2 cells. Renilla luciferase was used as an internal control. Luciferase activity was measured following the instructions of the dual-luciferase reporter kit (Promega, Madison, WI).

### 2.8. ELISA assay

The IL-1β and IL-18 levels in the supernatant fluid of HK-2 cells was measured using commercial kits according to the instructions. IL-1β (product codes: H002; detection range: 0.5–100 ng/l; inter-batch difference rate: CV < 10%; intra-batch difference rate: CV < 12%); IL-18 (product codes: H015; detection range: 1–300 ng/l; inter-batch difference rate: CV < 10%; intra-batch difference rate: CV < 12%). The absorbance at 450 nm was measured with a microplate reader (Thermo Fisher Scientific Inc., Waltham, MA).

### 2.9. Measurement of lactate dehydrogenase (LDH) level

Cells (1 × 10^6^) were seeded into six-well cell culture plates for 12 h and after treatment, the cell supernatant was collected. LDH levels were measured using an LDH assay kit (C0016, Beyotime, Shanghai, China) based on the manufacturer’s instructions. The absorbance was detected at 490 nm using a microplate reader (Thermo Fisher Scientific Inc., Waltham, MA).

### 2.10. qRT-PCR analysis

Total RNA was isolated using Trizol reagent (15596018, Ambion, Austin, TX) and stored at −80 °C for further detection. For mRNA analysis, cDNA was synthesized using the SuperScript™ IV First-Strand Synthesis System (6210A, Takara, Otsu, Japan), and qPCR was done using SYBR Green PCR Mastermix (SR1110, Solarbio, Beijing, China). For miRNA analysis, cDNA was synthesized using the Hairpin-it™ Real-Time PCR kit (E22001, GenePharma, Shanghai, China), which was analyzed using the corresponding U6 snRNA Normalization RT-qPCR Quantification Kit (E20001, GenePharma, Shanghai, China). GADPH was served as internal reference for mRNA analysis and U6 served as internal reference for miRNA analysis. Data were analyzed using by the 2^−△△Ct^ method. The primer sequences are listed in Table 1.

**Table 1.** Primer information.

	Forward primers	Reverse primers
IL-1β	5′-CCCAAAGTGGAAGATGGAAA-3′	5′-GGGTACAGGGCAGATTCAAA-3′
IL-18	5′-TGGCTGCTGAACCAGTAGAA-3′	5′-ATAGAGGCCGATTTCCTTGG-3′
miR-30e-5p	5′-GGGTGTAAACATCCTTGAC-3′	5′-TGCGTGTCGTGGAGTC-3′
ELAVL1	5′-GAGGCTCCAGTCAAAAACCA-3′	5′-GTTGGCGTCTTTGATCACCT-3′
PFKFB2	5′-AGTCCTACGACTTCTTTCGGC-3′	5′-TCTCCTCAGTGAGATACGCCT-3′
MAT2B	5′-ACAGAGAGGAAGACATACCAG-3′	5′-GTTCATTGCCAGACCAGTG-3′
HIPK1	5′-TCCCGCCTAAGCAGTGAAAAT-3′	5′-GGCAGGTATGATTCTTGTGCTG-3′
BCL2L11	5′-CACCAGCACCATAGAAGAA-3′	5′-ATAAGGAGCAGGCACAGA-3′
GAPDH	5′-AGGTCGGTGTGAACGGATTTG-3′	5′-GGGGTCGTTGATGGCAACA-3′
U6	5′-GCTTCGGCAGCACATATACTAAAAT-3′	5′-GCTTCGGCAGCACATATACTAAAAT-3′

### 2.11. Western blot analysis

Total protein extracts from HK-2 cells following various treatments were prepared in RIPA buffer containing protease inhibitors. The protein concentration was measured using a BCA assay kit (P0009, Beyotime, Shanghai, China). Thirty micrograms protein samples were separated by 12% sodium dodecyl sulfate-polyacrylamide gel electrophoresis (SDS-PAGE) and transferred to polyvinylidene fluoride membranes (Cytiva, Boston, MA). The membranes were blocked in TBS buffer containing 5% skim milk for 1 h and incubated with primary antibodies at 4 °C overnight. The membranes were washed with TBST and incubated with the corresponding secondary antibody for 1 h. The signal was detected using an ECL Kit (Thermo Fisher, Waltham, MA). Anti-CD63 (ab134045), anti-TSG101 (ab133586), anti-ELAVL1 (ab200342), anti-IL-1β (ab254360), anti-cleaved N-terminal domain of gasdermin D (GSDMD-N, ab215203), and anti-caspase-1 (ab207802) were obtained from Abcam (Cambridge, UK), Anti-NLRP3 (#15101), anti-cleaved-caspase 8 (#9496), anti-cleaved-caspase 3 (#9664), anti-cleaved-caspase 9 (#20750), and anti-GAPDH (#5174) were obtained from CST (Boston, MA). Anti-receptor-interacting protein kinase 1 (RIPK1, PA5-20811), anti-RIPK3 (PA5-19956), anti-mixed lineage kinase domain-like protein (MLKL, PA5-43960), anti-Janus kinase 2 (JAK2, 702434), and anti-signal transducer and activator of transcription 3 (STAT3, 710077) were purchased from Invitrogen (Waltham, MA).

### 2.12. Statistical analysis

All experiments were conducted at least three times and the data were presented as the means ± standard deviations. The data were analyzed using a one-way ANOVA followed by the least significance difference test. A *p* value <.05 was considered statistically significant.

## 3. Results

### 3.1. Isolation, identification, and internalization of BMSC-exos

**Figure 1.**
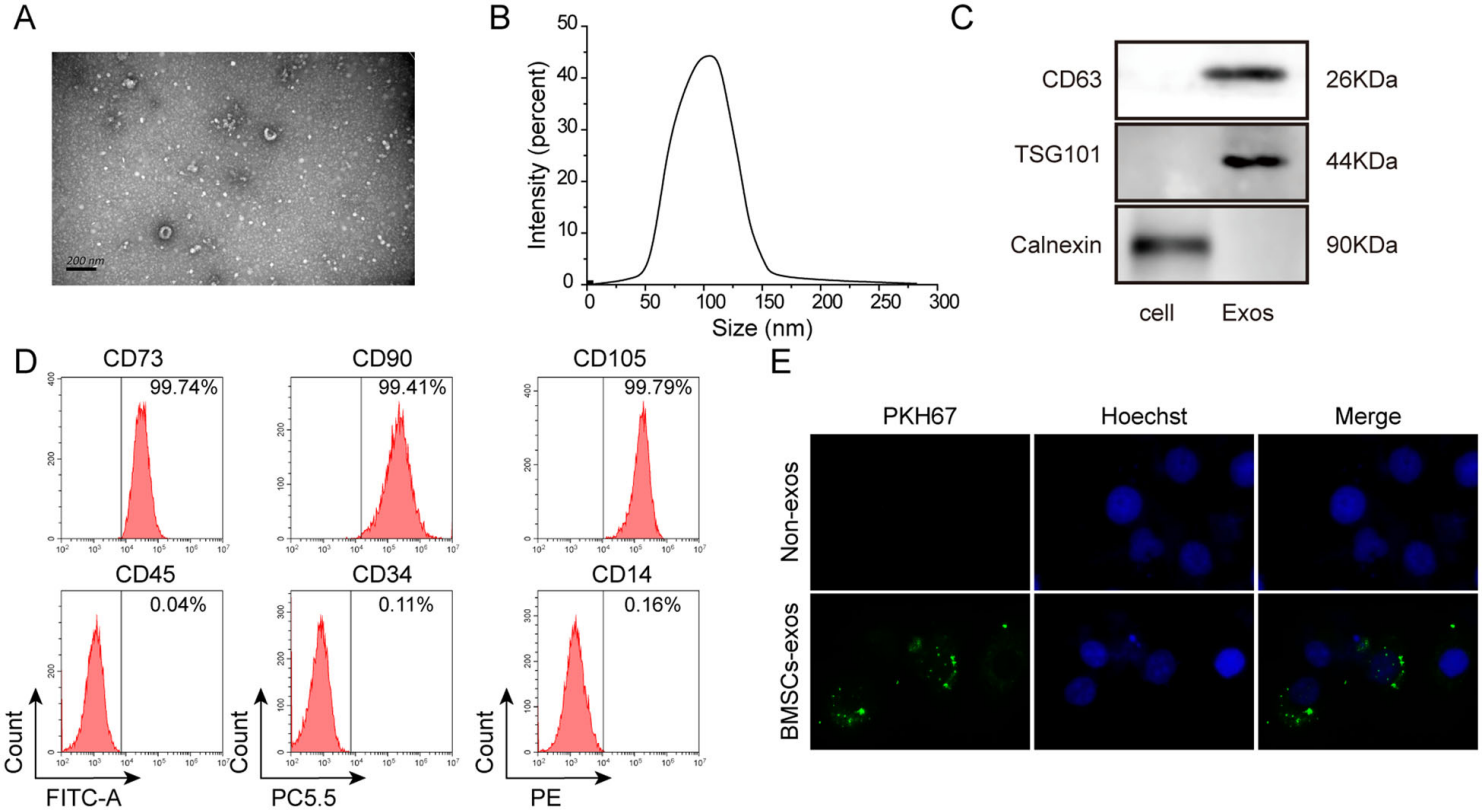
Isolation, identification, and internalization of exosomes derived from BMSCs. (A) Observation by transmission electron microscopy; (B) measurement of exosome size by nano-particle tracking molecule; (C) detection of BMSC-exos markers by western blot analysis; (D) detection of BMSC-exo markers by flow cytometry; (E) detection of labeled protein PKH67 by immunofluorescence assay. BMSC-exos: bone marrow MSC-derived exosomes. Three independent replications were performed.

The primary morphology of exosomes was ellipsoidal or cup-shaped as determined by transmission electron microscopy (Figure 1(A)). The size was measured by NTA and the diameter was ranged from 30 to 150 nm (Figure 1(B)). As shown in Figure 1(C), the positive markers, CD63 and TSG101 could be detected on BMSC-exos, whereas the negative marker calnexin was not detected in a western blot assay. Flow cytometry revealed that the exosomes were positive for CD73 (99.74%), CD90 (99.41%), and CD105 (99.79%), but negative for CD34 (0.11%), CD14 (0.16%), and CD45 (0.04%) (Figure 1(D)). The fluorescent dye PKH67 could stably bind to the lipid region of membranes and emit green fluorescence, thus enabling exosomes to be traced after co-culturing with cells. As shown in Figure 1(E), the marker protein PKH67, which was internalized into HK-2 cells, was successfully traced in the co-culture medium of BMSC-exos and HK-2 cells, indicating that BMSC-exos were absorbed by HK-2 cells. In summary, BMSC-exos were isolated, identified, and absorbed and internalized by HK-2 cells.

### 3.2. Effect of BMSC-exos on pyroptosis induced by HG

**Figure 2.**
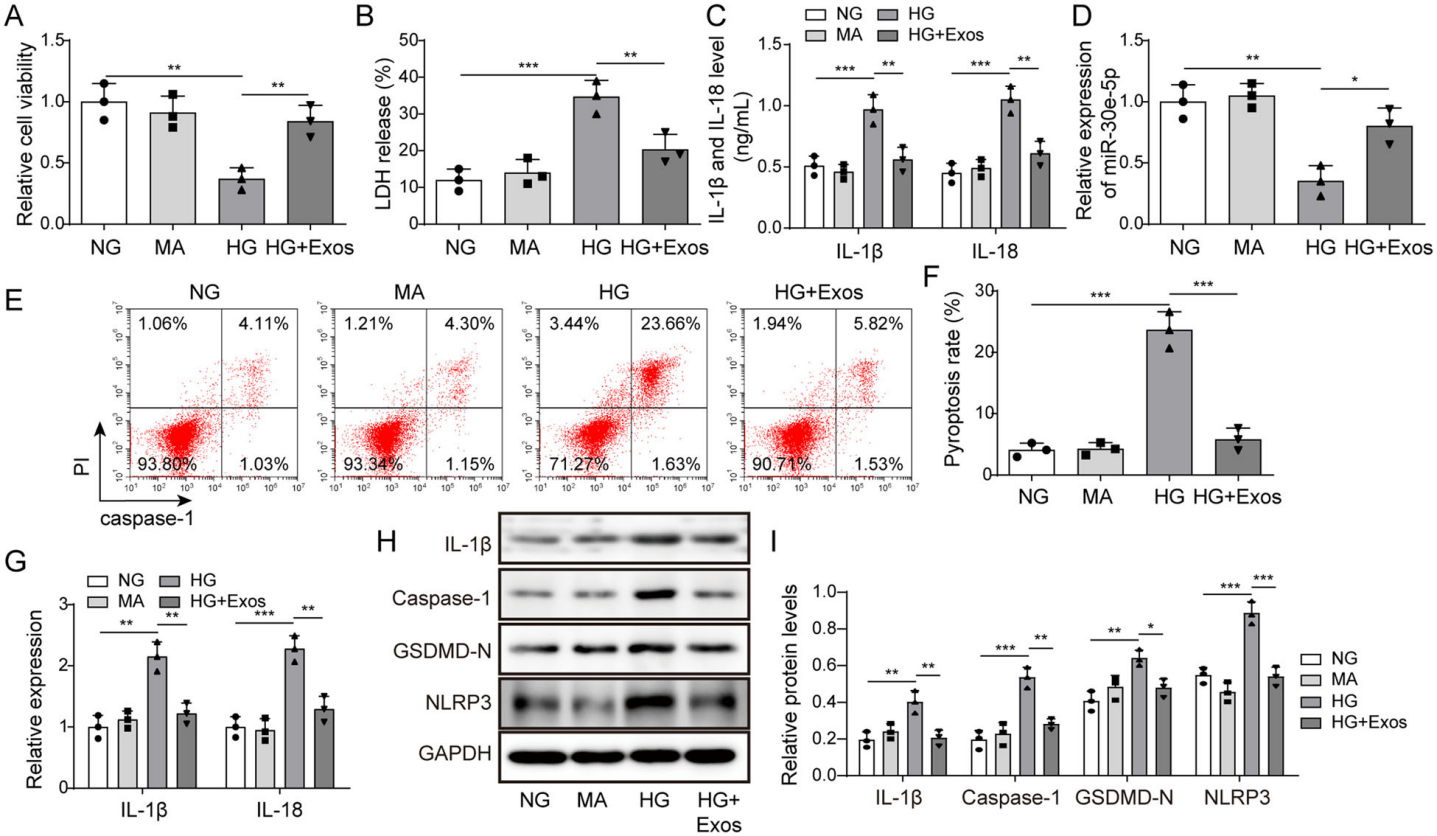
Effect of BMSC-exos on pyroptosis in HK-2 cells induced by HG. (A) The MTT assay was used to measure cell viability; (B) the LDH release assay was used to measure cytotoxicity; (C) IL-1β and IL-18 levels were measured by ELISA; (D) expression of miR-30e-5p measured by RT-qPCR; (E, F) pyroptosis levels were detected by flow cytometry; (G) IL-1β and IL-18 expression measured by qRT-PCR; (H, I) IL-1β, caspase-1, GSDMD-N, and NLRP3 expression measured by western blot analysis. Three independent replications were performed. *p<.05, **p<.01, and ***p<.001.

The role of BMSC-exos in pyroptosis induced by HG was evaluated. Cell viability was notably decreased in the HG group, but the inhibitory effect was reversed by BMSC-exos (Figure 2(A)). BMSC-exo treatment markedly decreased secreted LDH, IL-1β, and IL-18 levels in HG-treated cells (Figure 2(B,C)). MiR-30e-5p was highly expressed in the NG, MA, and HG + Exos groups, but the expression of miR-30e-5p in the HG group was decreased (Figure 2(D)). As shown in Figure 2(E,F), the level of pyroptosis was markedly decreased after BMSC-exos treatment in HG-induced cells. The expression of IL-1β and IL-18 mRNA in the HG group was up-regulated compared with the NG and MA groups, whereas they were significantly decreased in the HG + Exos group (Figure 2(G)). In addition, Figure 2(H,I) shows that the pyroptosis markers, GSDMD-N, IL-1β, NLRP3, and caspase-1, were markedly increased following HG treatment, but significantly decreased after BMSC-exo treatment. These results indicate that BMSC-exos ameliorate pyroptosis.

### 3.3. Effect of miR-30e-5p derived from BMSC-exos on pyroptosis

**Figure 3.**
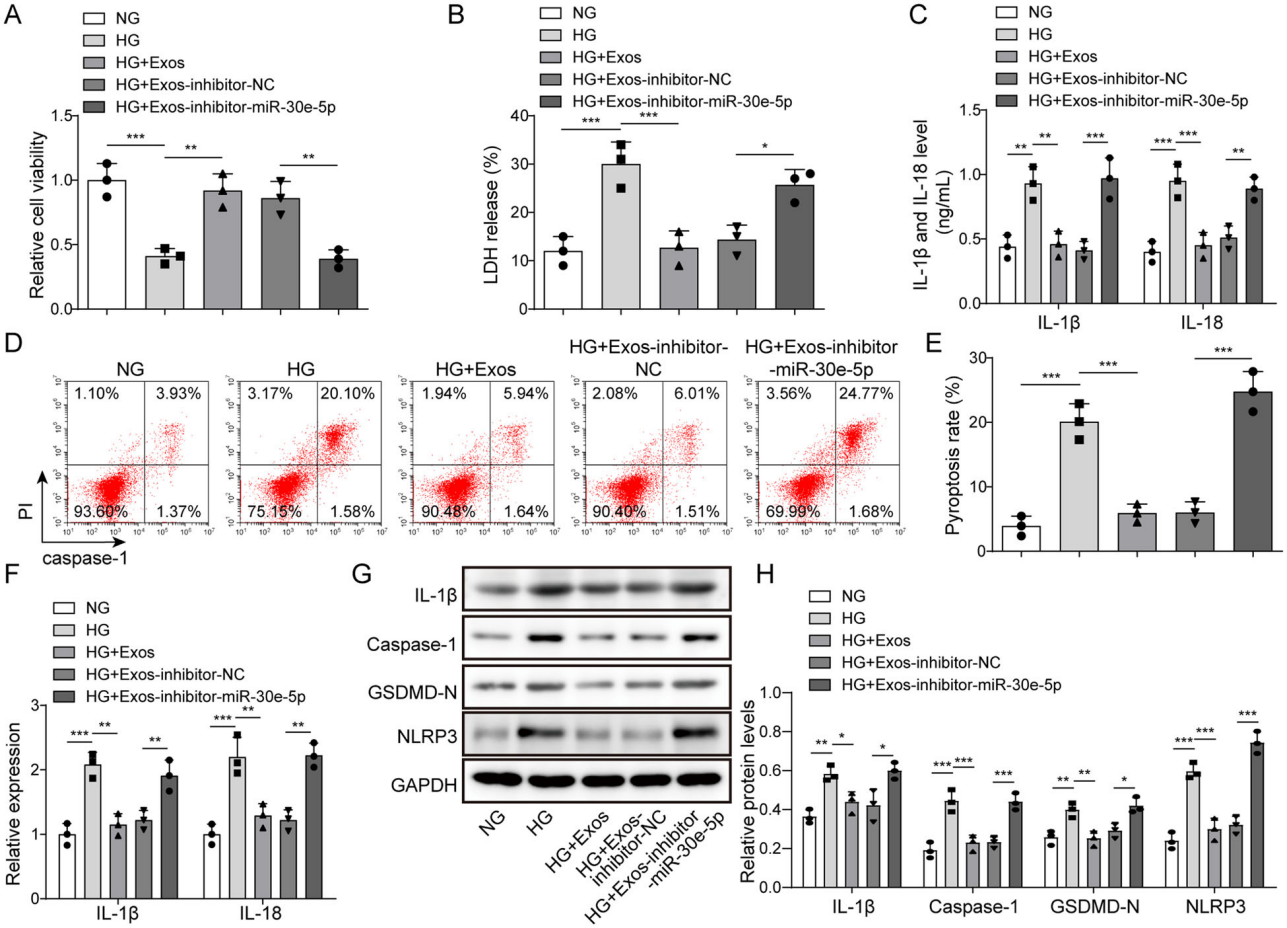
Effect of miR-30e-5p derived from BMSC-exos on pyroptosis. (A) The MTT assay was used to measure cell viability; (B) the LDH release assay was used to measure cytotoxicity; (C) IL-1β and IL-18 levels were measured by ELISA; (D, E) Pyroptosis levels as detected by flow cytometry; (F) IL-1β and IL-18 expression as measured by qRT-PCR; (G, H) IL-1β, caspase-1, GSDMD-N, and NLRP3 expression measured by western blot analysis. Three independent replications were performed. *p < .05, **p < .01, and ***p < .001.

The function of miR-30e-5p on pyroptosis was determined in cell transfection experiments. As shown in Figure 3(A), BMSC-exos increased cell viability in HG-induced cells, but the effect was eliminated by inhibiting miR-30e-5p. Similarly, the decreased release of LDH in BMSC-exo-treated cells was reversed by knocking down miR-30e-5p (Figure 3(B)). The release of inflammatory cytokines was markedly reduced by BMSC-exos, but reversed after inhibiting miR-30e-5p (Figure 3(C)). Moreover, the pyroptosis level in the HG + Exos-inhibitor-miR-30e-5p group was markedly increased compared with the HG + Exos-inhibitor-NC group (Figure 3(D,E)). Moreover, the expression of IL-1β and IL-18 was reduced by BMSC-exos in the HG treated cells and increased after miR-30e-5p inhibition (Figure 3(F)). The levels of GSDMD-N, IL-1β, NLRP3, and caspase-1 protein were also up-regulated after inhibiting miR-30e-5p (Figure 3(G,H)). The effect of miR-30e-5p derived from BMSC-exos on HG-induced necrosis was also determined by measuring receptor-interacting protein kinase 1 (RIPK1), p-RIPK1, receptor-interacting protein kinase 3 (RIPK3), p-RIPK3, and mixed lineage kinase domain-like protein (MLKL). As shown in Figure S1A and B, HG treatment up-regulated the necrosis-related proteins, p-RIPK1, p-RIPK3, and MLKL. The increase in these proteins was significantly inhibited by BMSC-exos treatment; however, further knockdown of miR-30e-5p reversed the down-regulating effect of BMSC-exos. The effect of miR-30e-5p derived from BMSC-exos on apoptosis and JAK2/STAT3 signaling is shown in Figure S1C and D. HG treatment up-regulated apoptosis-related proteins cleaved-caspase 8, cleaved-caspase 3, and cleaved-caspase 9, and activated the JAK2/STAT3 signal pathway. BMSC-exo treatment significantly inhibited the effect of HG treatment and further knockdown of miR-30e-5p reversed the down-regulation effect of BMSC-exos. Thus, miR-30e-5p derived from BMSC-exos exhibits a protective role in HG-induced pyroptosis, necrosis, apoptosis, and JAK2/STAT3 signaling in HK-2 cells.

### 3.4. Effect of miR-30e-5p overexpression or ELVAL1 knockdown on pyroptosis

**Figure 4.**
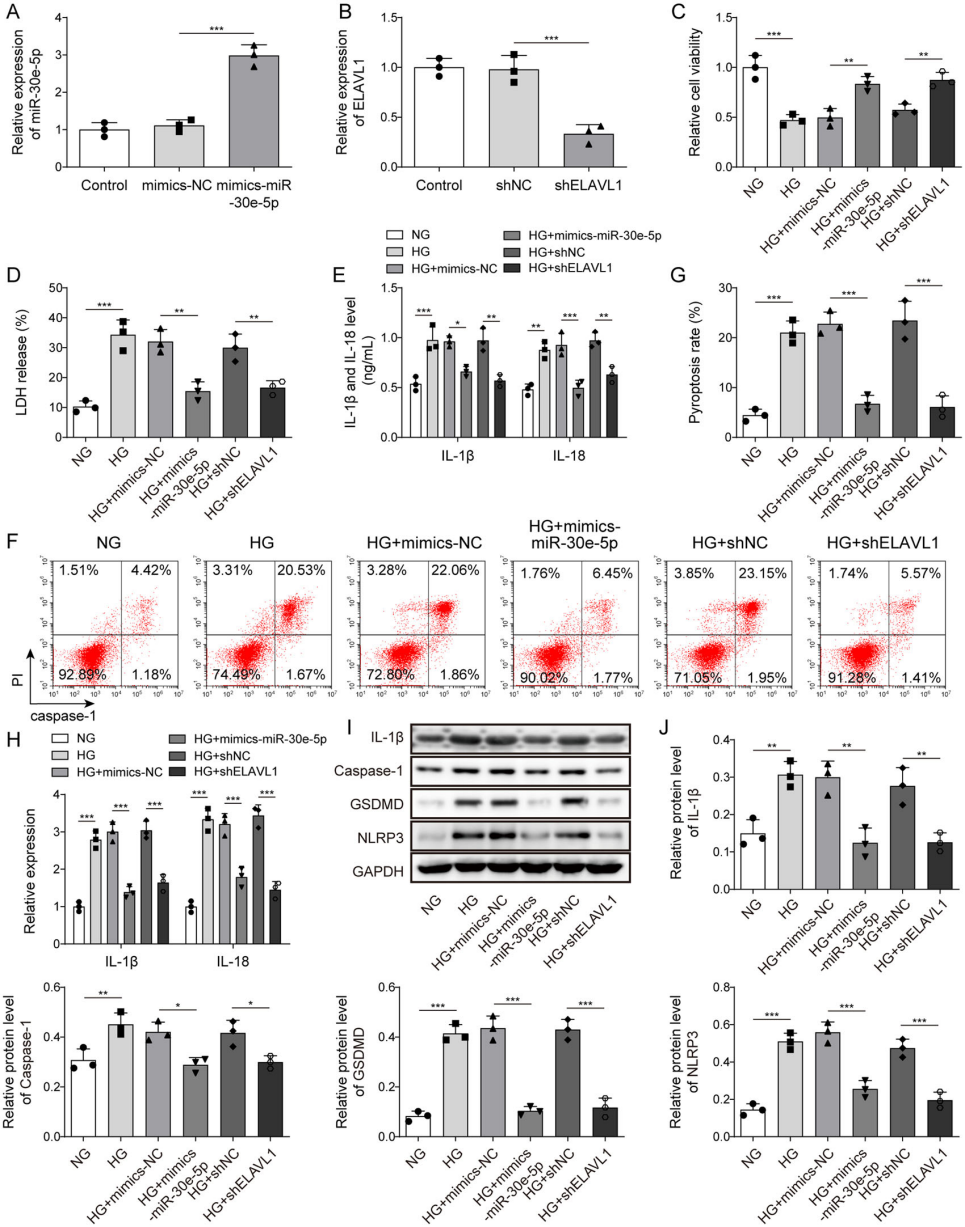
Effect of miR-30e-5p overexpression or ELVAL1 knockdown on HG-induced pyroptosis in HK-2 cells. (A) miR-30e-5p expression as measured by qRT-PCR; (B) ELVAL1 expression as measured by qRT-PCR; (C) the MTT assay was used to measure cell viability; (D) the LDH release assay was used to measure cytotoxicity; (E) IL-1β and IL-18 levels were measured by ELISA; (F, G) pyroptosis levels as detected by flow cytometry; (H) IL-1β and IL-18 expression as measured by qRT-PCR; (I, J) IL-1β, caspase-1, GSDMD-N, and NLRP3 expression as measured by western blot analysis. Three independent replications were performed. *p < .05, **p < .01, and ***p < .001.

As demonstrated from the above results, miR-30e-5p derived from BMSC-exos inhibited pyroptosis in HG-induced HK-2 cells. What’s more, as ELAVL1 played important role in activating pyroptosis. We then investigated the effects of miR-30e-5p overexpression or ELVAL1 knockdown on HG-induced pyroptosis. The results of Figure 4(A,B) indicated that miR-30e-5p and ELVAL1 were successfully overexpressed and knocked down, respectively. Figure 4(C) shows that HG treatment inhibited cell viability; however, miR-30e-5p overexpression or ELVAL1 knockdown promoted cell viability. In addition, LDH release in the HG group was significantly higher compared with that in the NG group, whereas overexpression of miR-30e-5p or knockdown of ELVAL1 down-regulated LDH release (Figure 4(D)). Similarly, up-regulated secretion of IL-1β and IL-18 by HG was reversed by miR-30e-5p overexpression or ELVAL1 knockdown (Figure 4(E)). Flow cytometry revealed that HG promoted pyroptosis, which was abolished by miR-30e-5p overexpression or ELVAL1 knockdown (Figure 4(F,G)). Moreover, miR-30e-5p overexpression or ELVAL1 knockdown reversed the up-regulation effect on IL-1β and IL-18 expression induced by HG (Figure 4(H)). Finally, HG was increased the levels of pyroptosis markers, such as IL-1β, caspase-1, GSDMD, and NLRP3; however, overexpression of miR-30e-5p or knockdown of ELVAL1 significantly down-regulated pyroptosis-related proteins (Figure 4(I,J)). These results indicated that miR-30e-5p overexpression or ELVAL1 knockdown directly inhibited pyroptosis.

### 3.5. Targeting effect of miR-30e-5p and ELAVL1

**Figure 5.**
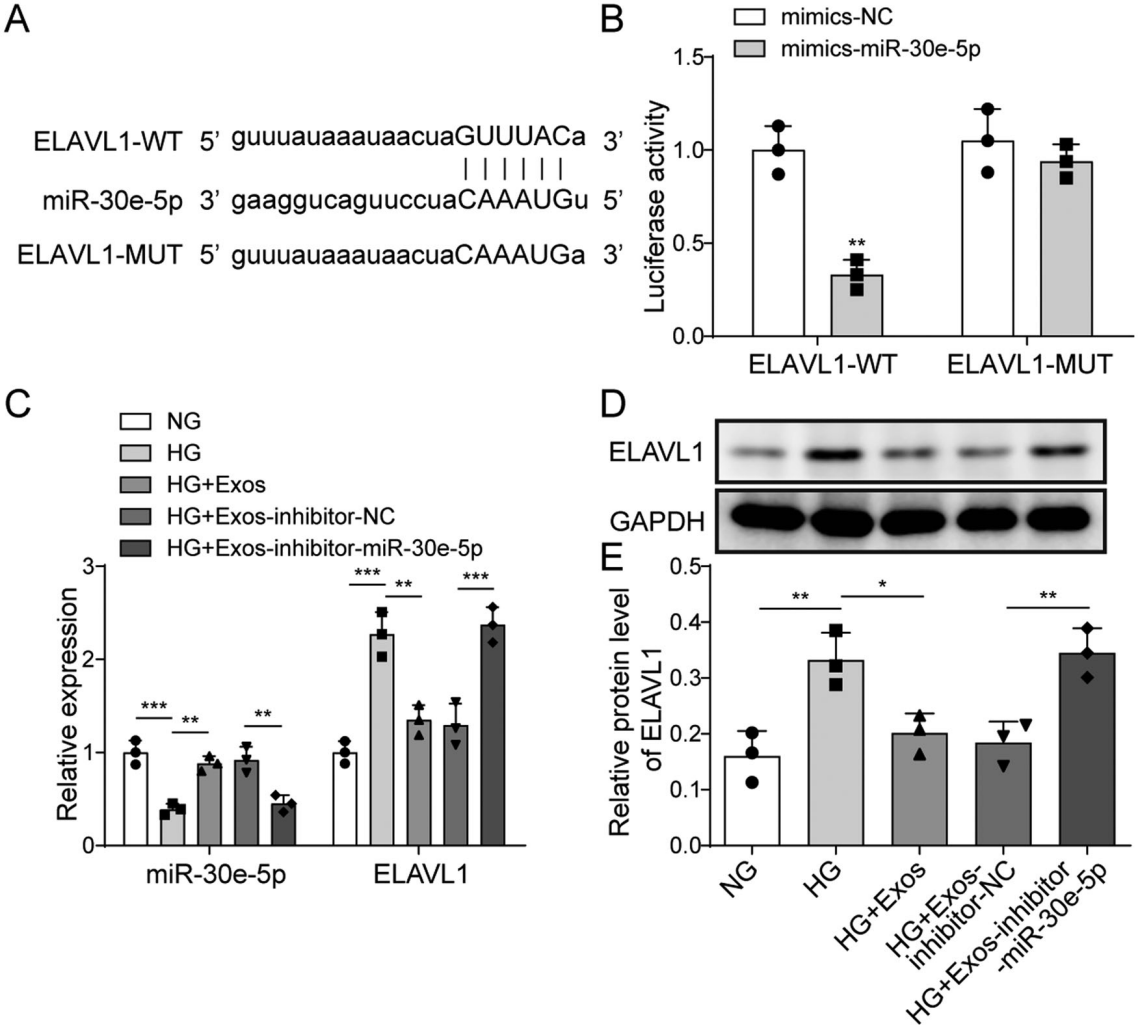
Targeting effect of miR-30e-5p and ELAVL1. (A) Binding site of miR-30e-5p and ELAVL1 predicted by starBase; (B) dual-luciferase reporter assay measuring the binding relationship between miR-30e-5p and ELAVL1; (C) MiR-30e-5p and ELAVL1 expression as measured by qRT-PCR; (D, E) expression of ELAVL1 as measured by western blot analysis. Three independent replications were performed. *p < .05, **p < .01, and ***p < .001.

Using the starBase database, we found that miR-30e-5p might bind to a large number of genes including ELAVL1 (Figure 5(A)). We tested several genes that we were interested in and the results indicated that HG treatment up-regulated ELAVL1 and methionine adenosyltransferase 2B (MAT2B) mRNA levels, down-regulated 6-phosphofructo-2-kinase/fructose-2 (PFKB2) levels, but had no effect on homeodomain interacting protein kinase 1 (HIPK1) and Bcl2-like protein 11 (BCL2L11) levels (Fig. S2). Among them, the change of ELAVL1 level was the most pronounced following HG treatment. To verify the targeting effect between miR-30e-5p and ELAVL1, a dual-luciferase reporter assay was used. We found that miR-30e-5p mimics down-regulated luciferase activity in cells when co-transfected with wt-ELAVL1 (Figure 5(B)). Thus, a mutual binding relationship existed between ELAVL1 and miR-30e-5p. In addition, qRT-PCR revealed that ELAVL1 expression was up-regulated after miR-30e-5p inhibition (Figure 5(C)). Consistently, ELAVL1 protein expression was up-regulated after inhibiting miR-30e-5p (Figure 5(D,E)). These results suggest that ELAVL1 is a target of miR-30e-5p.

### 3.6. Effect of miR-30e-5p/ELAVL1 axis on pyroptosis

**Figure 6.**
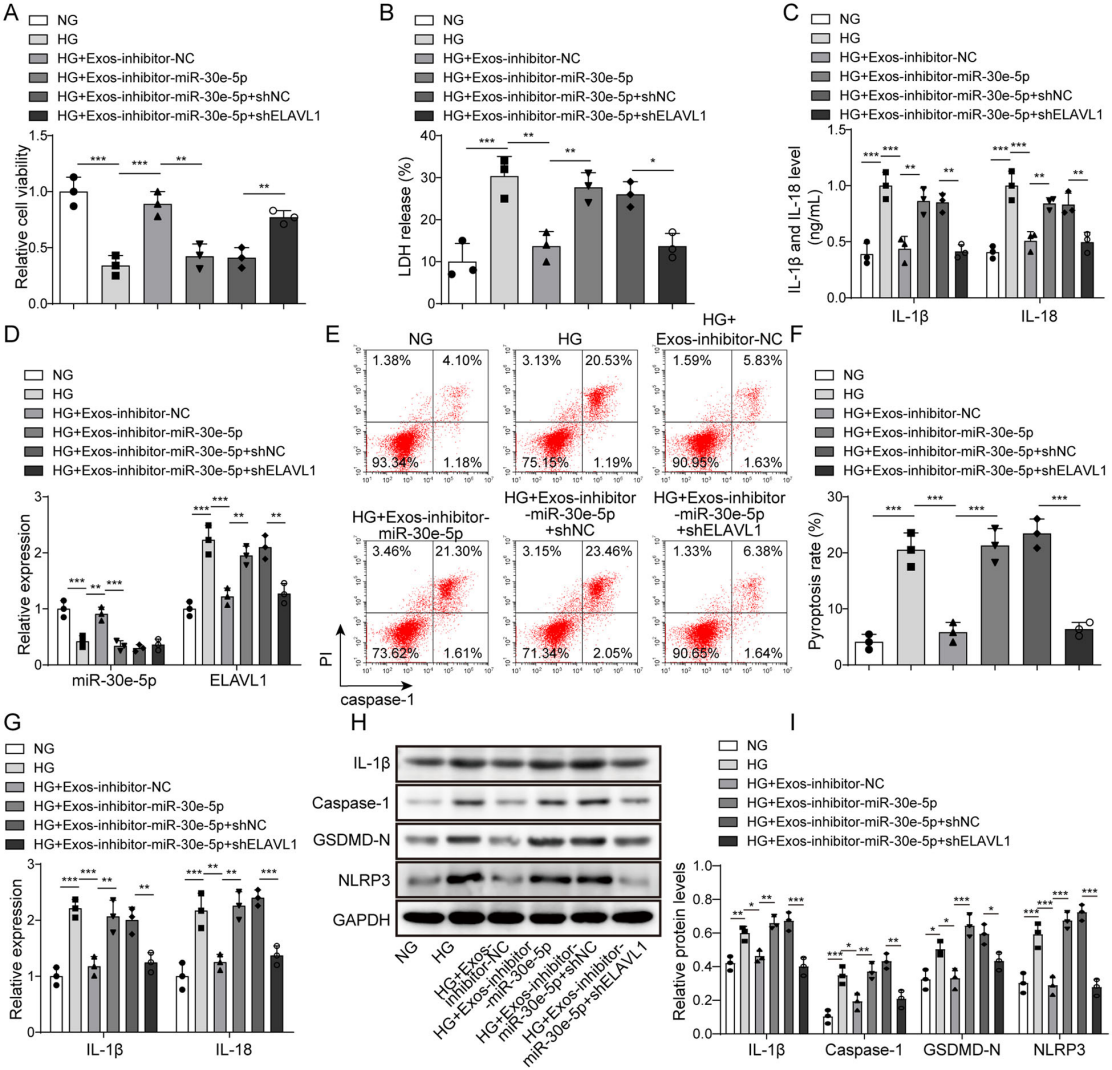
Effect of the miR-30e-5p/ELAVL1 axis on pyroptosis. (A) The MTT assay was used to measure cell viability; (B) the LDH release assay was used to measure cytotoxicity; (C) IL-1β and IL-18 levels were measured by ELISA; (D) expression of miR-30e-5p and ELAVL1 as measured by qRT-PCR; (E, F) pyroptosis levels as detected by flow cytometry; (G) IL-1β and IL-18 expression as measured by qRT-PCR; (H, I) IL-1β, caspase-1, GSDMD-N, and NLRP3 expression as measured by western blot analysis. Three independent replications were performed. *p < .05, **p < .01, and ***p < .001.

To confirm the effect of miR-30e-5p on pyroptosis regulated by ELAVL1, a BMSC-exo-treated diabetes model was established. Cell viability was notably decreased when inhibiting miR-30e-5p, whereas knocking down ELAVL1 reversed this effect (Figure 6(A)). The release of LDH was increased by inhibiting miR-30e-5p, but decreased when ELAVL1 was knocked down (Figure 6(B)). In addition, the levels of secreted IL-1β and IL-18 were increased by inhibiting miR-30e-5p and decreased again after knocking down ELAVL1 (Figure 6(C)). The level of miR-30e-5p following knockdown remained under-expressed after knocking down sh-ELAVL1. Nevertheless, the high expression of ELAVL1 caused by miR-30e-5p knockdown was down-regulated by sh-ELAVL1 (Figure 6(D)). The level of pyroptosis was increased following miR-30e-5p inhibition, whereas this effect was reversed by knocking down ELAVL1 (Figure 6(E,F)). In addition, the promoting effects of the miR-30e-5p inhibitor on IL-1β and IL-18 expression were dramatically reversed by ELAVL1 knockdown in the BMSC-exo-treated diabetes model cells (Figure 6(G)). Similarly, the levels of GSDMD-N, IL-1β, NLRP3, and caspase-1 were also down-regulated in the HG + Exos-miR-30e-5p inhibitor + shELAVL1 group (Figure 6(H,I)). These results indicate that BMSC-exos may transfer miR-30e-5p into HK-2 cells, which ameliorates HG-induced pyroptosis by inhibiting ELAVL1.

## 4. Discussion

Both type I and type II diabetes can induce DKD [28]. However, despite extensive efforts in disease control, many patients develop not only early- and middle-stage DKD, but also end-stage chronic kidney disease. The current treatments remain limited and novel therapeutics are needed to control the progression of this disease. In the present study, BMSC-exos were shown to alleviate HG-induced HK-2 cell pyroptosis. Furthermore, BMSC-derived exosomal miR-30e-5p ameliorated HK-2 cell pyroptosis by modulating ELAVL1.

In the past few decades, MSCs have gained interest as a potential therapy for DKD. MSCs have many physiological functions including self-renewal, clone cell population generation, and multi-line differentiation [29]. As a treatment for DKD, MSCs were shown to reduce hyperglycemia and proteinuria, as well as improve renal pathological changes [30–32]. BMSC-exos can serve as vehicles to treat DKD based on their ability to exchange bioactive components and transport genetic contents [33]. In the present study, we demonstrated that BMSC-exos ameliorate pyroptosis in HG-induced HK-2 cells. Our findings provided an alternative strategy for the treatment of DKD, although the putative mechanism needs clarification.

Several studies have shown that various miRNAs are associated with DKD [31,32]. For example, miR-185 was reported to regulate the deposition of the DKD extracellular matrix by targeting TGF-β1 [34]. The expression of miR-497 was down-regulated in the kidneys of mice with diabetes [35]. As an emerging regulator of disease progression, exosomal miRNAs are associated with a class of metabolic disorders, including diabetes and its complications [22]. Exosomal miR-215-5p derived from adipose-derived stem cells was protective against migration and inhibited podocyte damage in DKD [22]. Furthermore, exosomal miR-19b-3p derived from tubular epithelial cells inhibited inflammation to regulate DKD progression [23]. To our knowledge, miR-30e-5p has an important role in diabetes. We demonstrated the beneficial effect of BMSC-exos on HG-induced HK-2 cell pyroptosis by transferring miR-30e-5p. This may also be helpful for using miR-30e-5p to regulate DKD.

Recent evidence has indicated that pyroptosis, a pro-inflammatory mode of programmed cell death, is involved in DKD [36]. Due to chronic exposure to diabetic substrates, pyroptosis and renal inflammation occur [37]. During the progression of pyroptosis, caspase-1 is activated by a large supramolecular complex known as the pyroptosome, which subsequently activates IL-1β and IL-18. In the present study, HG acted as a pyroptosis inducer. LDH, IL-1β, and IL-18 secretion was increased in the HG group as well as GSDMD-N, IL-1β, NLRP3, and caspase-1 expression. ELAVL1, a component of the pyroptosome involved in activating pyroptosis, promotes IL-1β and IL-18 secretion combined with caspase-1 activation [22]. Another important finding of this study was the binding relationship between ELAVL1 and miR-30e-5p. We found that miR-30e-5p could inhibit caspase-1-mediated pyroptosis in HG-induced HK-2 cells by targeting ELAVL1.

The nephron is the basic unit of kidney structure, including the renal body and connected renal tubules, which play an important role in regulating kidney function. The abnormal function of renal tubular epithelial cells is a key factor leading to DKD. Apoptosis of renal tubular epithelial cells results in abnormal renal tubular re-absorption and renal fibrosis [38]. HK-2 cells have most of the functional characteristics of normal adult renal proximal tubular cells, thus they are widely used to study renal pathology and screen therapeutic drugs *in vitro* [39]. This study focused on the regulatory mechanism of BMSC-exo-derived miR-30e-5p on HG-induced pyroptosis in HK-2 cells. The main pathological features of DKD include glomerular hypertrophy, increased production and accumulation of extracellular matrix, mesangial expansion, basement membrane thickening, glomerular follicular loss, and renal fibrosis, which leads to the progressive loss of renal function [40]. Whether miR-30e-5p affects these pathological changes in DKD *in vivo* needs to be confirmed. In addition, the glomerular filtration barrier consists of the basement membrane, the inner endothelium, and the outer podocyte epithelium. DKD was shown to be associated with necrosis of podocytes and mesangial cells [41,42]. Moreover, high glucose (HG) levels inhibited autophagy by activating the JAK/STAT pathway of podocytes, thereby preventing the effective removal of damaged proteins and organelles from the body to prevent apoptosis, and ultimately, aggravating podocyte injury and DKD progression [43]. Glomerular endothelial cell injury is thought to contribute to the development of microalbuminuria, an early event in DKD [44]. In addition, recent studies suggested that glomerular endothelial cells may contribute to DKD through paracrine communication with other glomerular cells, such as podocytes and mesangial cells. Therefore, in addition to tubular epithelial cells, mesangial cells, and podocytes also play an important role in apoptosis, necrosis, and autophagy in DKD. However, whether BMSC-exo-derived miR-30e-5p regulates apoptosis, pyroptosis, or the necrotizing apoptosis process of other renal cells or downstream signaling warrants further investigation.

In conclusion, our findings demonstrate the ameliorative effect of BMSC-derived exosomal miR-30e-5p on HG-induced HK-2 cell pyroptosis. The underlying mechanism involves the inhibitory effect of miR-30e-5p on ELAVL1 and caspase-1-mediated pyroptosis. However, our work lacks clinical and animal experiments for further validation. In subsequent studies, we will certainly explore the effects of BMSCs-exos on the development of DKD *in vivo*. Our study is expected to provide new target and direction for the therapy of DKD.

## Supplementary Material

Supplemental Material

Supplemental Material
