# Supplementary figures and images for: Bone marrow mesenchymal stem cell-derived exosomal miR-30e-5p ameliorates high-glucose induced renal proximal tubular cell pyroptosis by inhibiting ELAVL1
Source: Ren Fail. 2023 Feb 16;45(1):2177082. doi: 10.1080/0886022X.2023.2177082 (PMC9937013; doi:10.1080/0886022X.2023.2177082)

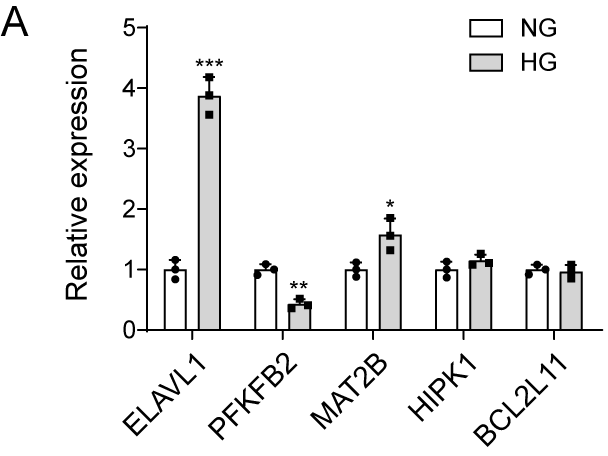

Supplement: Supplemental Material [file IRNF_A_2177082_SM0017.tif]

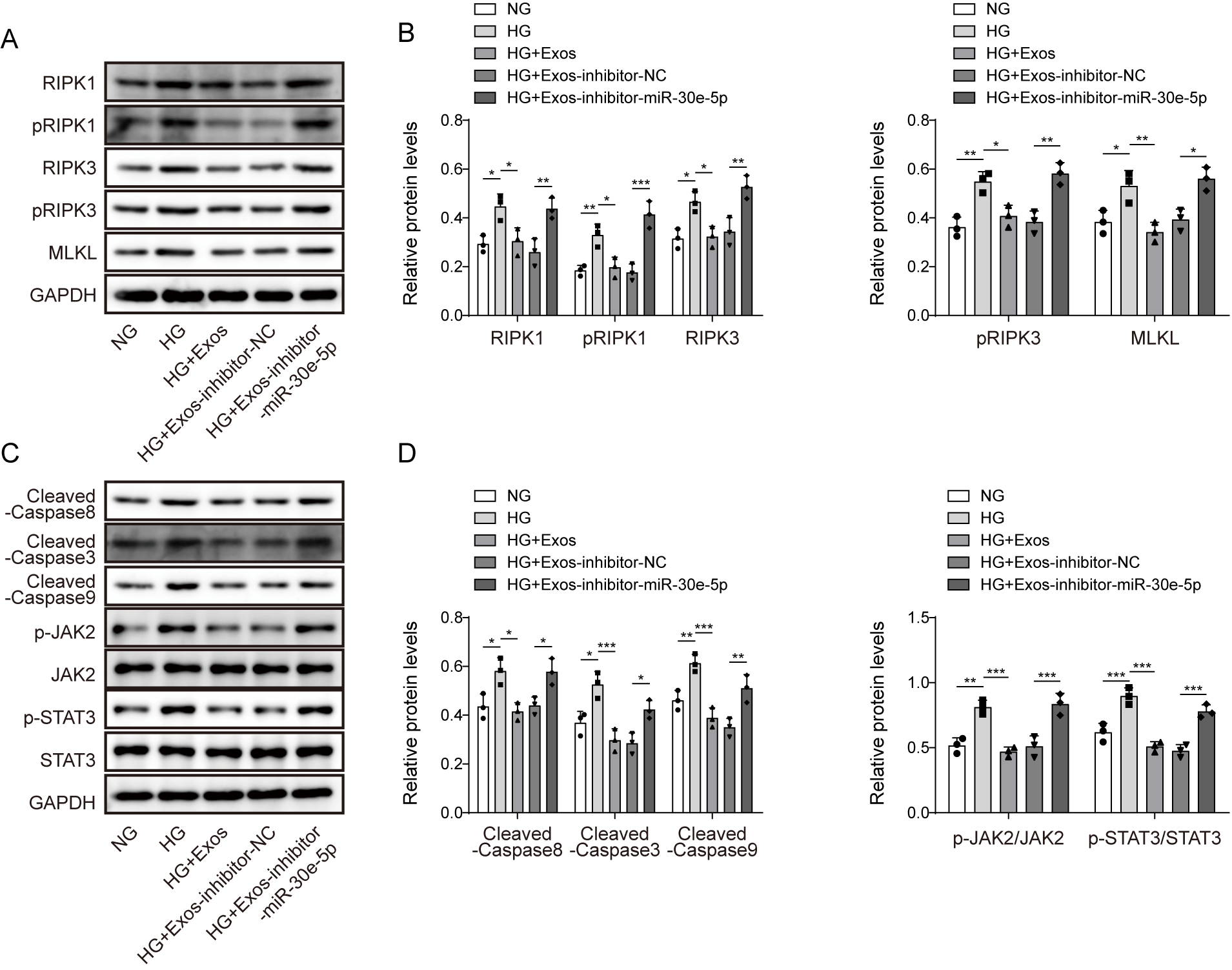

Supplement: Supplemental Material [file IRNF_A_2177082_SM0010.tif]
